# ​Bullet to the Heart: A Case Report

**DOI:** 10.7759/cureus.84268

**Published:** 2025-05-17

**Authors:** Gustavo Christian-Colón, Gabriel Figueroa-Martínez, Derick Rodriguez-Reyes, Raysa López-Alvarado, Nelimar Cruz-Centeno, Nydia De Soto-Cordero

**Affiliations:** 1 Surgery, Ponce Health Sciences University, Ponce, PRI; 2 General Surgery, University of Puerto Rico, Medical Sciences Campus, San Juan, PRI; 3 Emergency Medicine, University of Puerto Rico, Medical Sciences Campus, San Juan, PRI; 4 Trauma Surgery, University of Puerto Rico, Medical Sciences Campus, San Juan, PRI

**Keywords:** acute care surgery and trauma, cardiac trauma, gunshot wound, puerto rico, surgical case reports

## Abstract

Penetrating cardiac injuries (PCI) from gunshot wounds are among the most fatal forms of trauma, with prehospital mortality rates exceeding 90%. While the right ventricle is most commonly affected due to its anterior location, retained intracardiac projectiles are rarely encountered and pose significant management challenges. Cardiac tamponade, though potentially fatal, can sometimes provide a protective mechanism by limiting hemorrhage. The decision to surgically remove retained bullets remains controversial, particularly in hemodynamically stable patients. We report the case of a 51-year-old male who sustained multiple gunshot wounds, including a thoracic injury with an 8 mm bullet fragment retained in the right ventricle. On arrival, the patient was hemodynamically stable despite imaging revealing a pericardial effusion and a sternal fracture. A median sternotomy with pericardiotomy was performed, revealing a right ventricular epicardial wound without active bleeding. Due to the absence of cardiopulmonary bypass, the intracardiac bullet was not removed. The patient received prophylactic heparin and was monitored with imaging and serial exams. He was discharged without complications. This case highlights a rare presentation of PCI without overt tamponade physiology, despite the presence of a pericardial effusion. The patient remained stable throughout hospitalization, supporting the hypothesis that tamponade can temporarily contain hemorrhage. Conservative management of retained intracardiac projectiles may be appropriate in select patients, particularly when the projectile is embedded in the trabeculated myocardium and the risk of embolization is low. Literature suggests that long-term outcomes can be favorable with nonoperative management in stable cases. This case reinforces the importance of rapid surgical intervention for PCI, even in stable patients, and supports individualized, conservative management of retained cardiac projectiles in the absence of cardiopulmonary bypass. Further research is needed to define standardized protocols for managing intracardiac foreign bodies in trauma.

## Introduction

Penetrating cardiac injuries (PCI), particularly those resulting from gunshot wounds (GSWs), represent one of the most lethal forms of trauma and pose a significant challenge to trauma and cardiothoracic teams. These injuries are rare, with an incidence of only 0.16% among trauma admissions in the United States [1]. However, they are associated with a disproportionately high mortality rate, with over 90% of patients dying before reaching the hospital due to exsanguination or cardiac tamponade [2-4]. For those who do survive to hospital admission, survival rates vary widely - from 3% to 84% - depending on various factors, such as hemodynamic stability, the location and severity of the injury, and the presence of associated trauma [5-7].

Most PCIs occur in young males and are frequently the result of interpersonal violence. The right ventricle is the most affected cardiac chamber due to its anterior anatomical position within the mediastinum [8]. While advances in trauma systems and operative management have improved outcomes, PCI remains a time-sensitive surgical emergency requiring rapid assessment and intervention. Cardiac tamponade, although potentially fatal, may paradoxically act as a protective mechanism by slowing exsanguination through increased pericardial pressure [9].

There is limited literature addressing the management of retained intracardiac projectiles, particularly in the absence of cardiopulmonary bypass (CPB). Some reports advocate for immediate surgical removal due to the risk of embolization or valvular injury, while others support conservative management in asymptomatic, hemodynamically stable patients [10-12]. Our case contributes to this discussion by presenting a rare instance of a bullet lodged in the right ventricle, managed without CPB due to resource limitations. The patient remained clinically stable postoperatively, and the projectile was retained with no signs of embolic complications. This report adds to the limited body of evidence guiding decision-making in such cases and supports individualized, context-sensitive management.

## Case presentation

 A 51-year-old male with a history of hypertension presented to the trauma bay at a level 2 trauma center after sustaining multiple gunshot wounds (GSWs). He had no known drug allergies and was on lisinopril and hydrochlorothiazide. His past surgical history was notable only for laparoscopic umbilical hernia repair 10 years prior. The patient sustained two GSWs to the left hand, one to the left arm, one to the right flank, and two to the anterior chest. The incident occurred on December 9, 2024, at approximately 1:00 PM. He was initially evaluated and imaged at an outside facility and transferred to our trauma center at 5:00 PM for definitive management.

Upon arrival, the patient was alert, oriented, and hemodynamically stable. He reported substernal chest pain that was sharp, non-radiating, and exacerbated by inspiration. His initial vital signs included a temperature of 37.1°C, heart rate of 105 bpm, blood pressure of 112/75 mmHg, respiratory rate of 24 breaths/min, and oxygen saturation of 100% on a 50% Venturi mask. On physical exam, he had a patent airway and was speaking in full sentences. Chest exam revealed symmetric rise with inspiration, no accessory muscle use, and clear breath sounds bilaterally. No intercostal retractions or evidence of subcutaneous emphysema was noted. Two gunshot entry wounds were identified on the chest - one at the lower left sternal border and one at the right parasternal border - both without active bleeding. Cardiac auscultation revealed regular rhythm with slightly distant heart sounds; no murmurs, rubs, or gallops were appreciated. There was no jugular venous distension (JVD), and no signs of Beck’s triad were present on initial survey.

Abdominal examination was benign aside from a single GSW entry wound to the right flank. The abdomen was soft, non-tender, and without peritoneal signs or ecchymosis. Peripheral pulses were strong and symmetric (2+ bilaterally). A focused assessment with sonography for trauma (FAST) exam was negative for intra-abdominal free fluid but revealed a small pericardial effusion. Examination of the left upper extremity revealed complex dorsal hand trauma with an open laceration exposing soft tissue and tendinous structures, irregular wound margins, active oozing, and associated swelling and ecchymosis. No signs of compartment syndrome or vascular compromise were present. The right upper extremity was intact.

**Figure 1:**
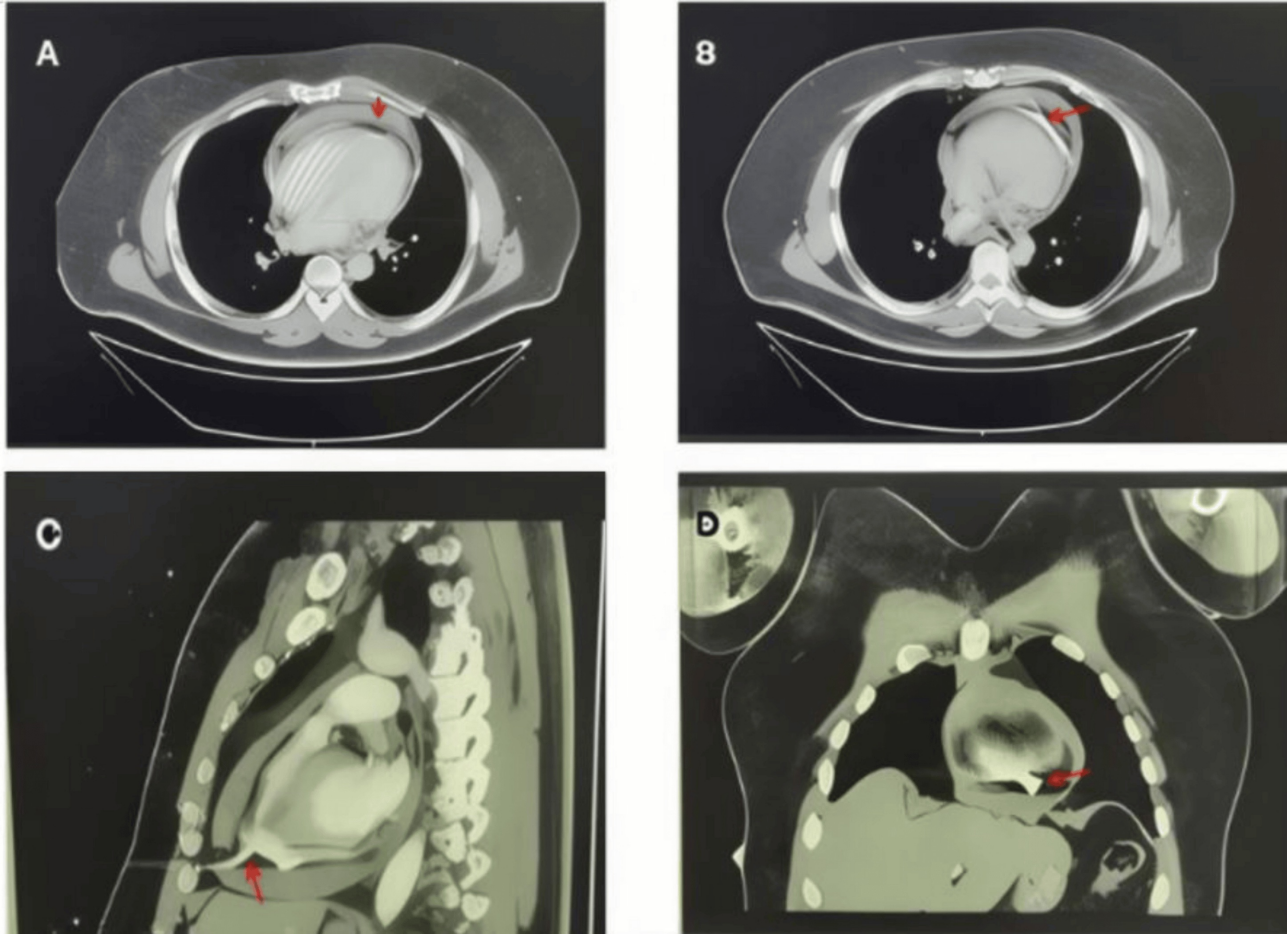
Computerized tomography (CT) of the chest brought from outside institution (A) and (B) are transverse showing pericardial effusion and retained bullet fragment, respectively, marked with red arrows. (C) is sagittal orientation showing a retained bullet fragment in the apical region marked by a red arrow. (D) is the coronal orientation scan showing the same findings. Red arrows used to mark pericardial effusion in A, bullet fragment in B, C, and D.

**Figure 2:**
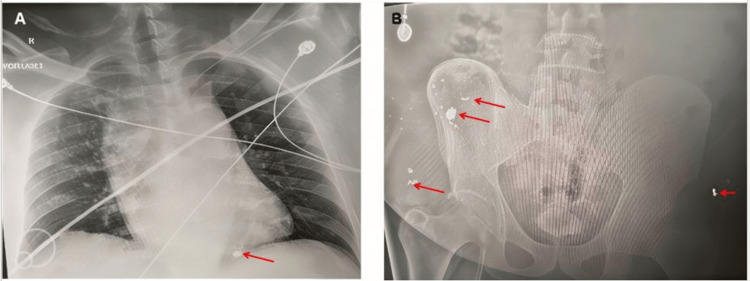
Radiographic imaging taken at trauma bay (A) Portable chest X-ray showing wide mediastinum and projectile lodged in heart, marked by a red arrow. (B) Pelvic X-ray showing the majority of bullet fragments projecting along the right wing of the ilium, labeled by red arrows. Red arrows on A and B pointing towards bullet fragments in chest X-ray and pelvic X-ray.

Imaging studies performed at the outside hospital included a chest CT scan, which showed a sternal body fracture, pericardial effusion, and an 8 mm bullet fragment embedded in the apical region of the right ventricle (Figure 1). No evidence of pneumothorax or hemothorax was observed. A portable chest X-ray taken in the trauma bay showed a widened mediastinum and retained cardiac projectile, while a pelvic X-ray revealed bullet fragments over the right iliac wing (Figure 2).

Laboratory studies demonstrated elevated creatine phosphokinase (1,988 U/L) and lactic acid (22.7 mg/dL), suggestive of tissue injury and early shock physiology. Troponins were within normal limits. Electrocardiogram (EKG) showed sinus tachycardia without ST-segment changes or electrical alternans. Given the imaging findings of pericardial effusion and retained cardiac projectile, the patient was taken emergently to the operating room for median sternotomy and pericardiotomy.

**Figure 3:**
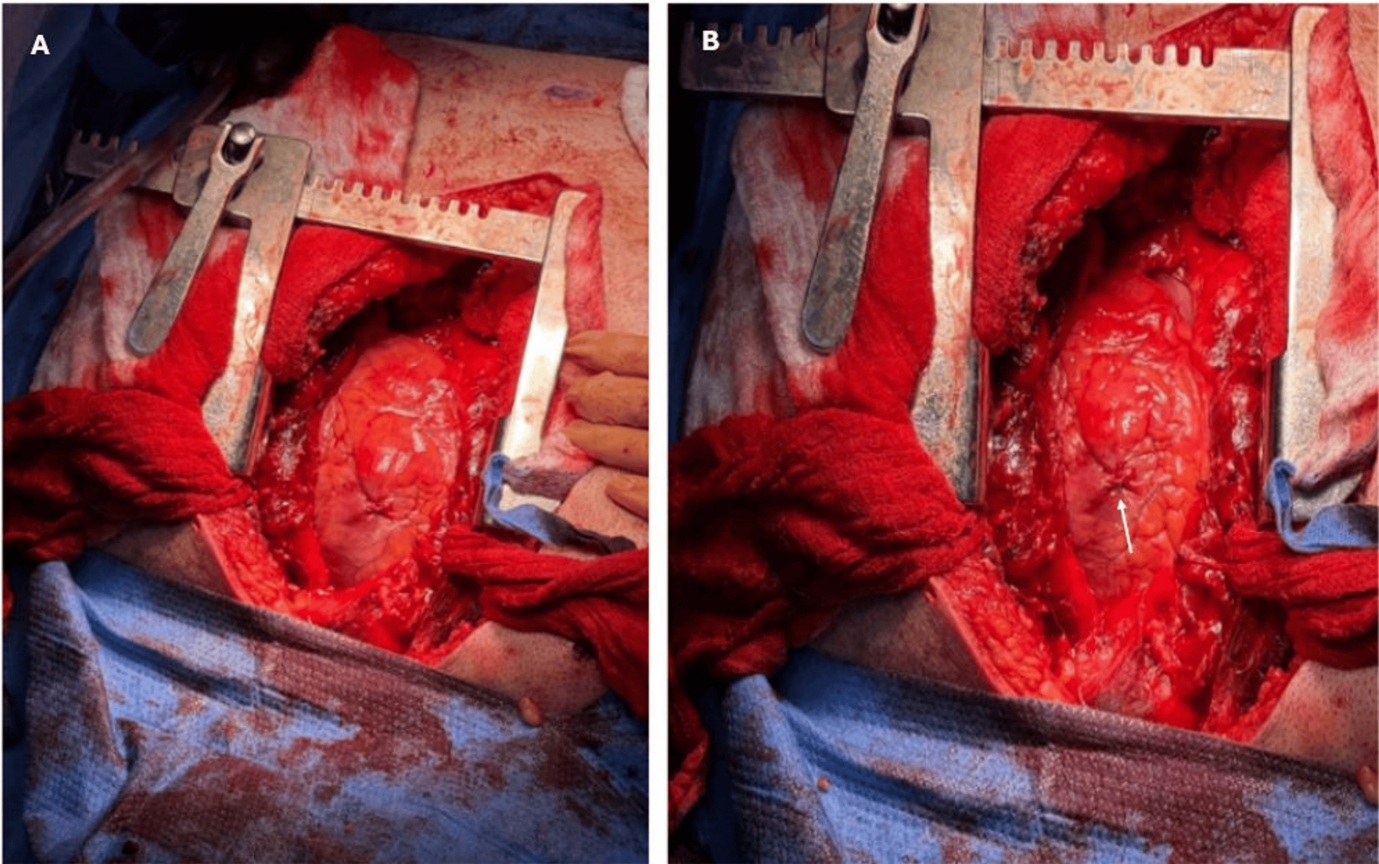
Intraoperative findings during median sternotomy (A) Penetrating cardiac injury visualized at the anterior superior wall of the right ventricle with no active bleeding from site. (B) Penetrating cardiac injury post primary repair with a suture marked by a white arrow. White arrow on B used to identify the region of the penetrating cardiac injury that was treated via suture primary repair.

**Figure 4:**
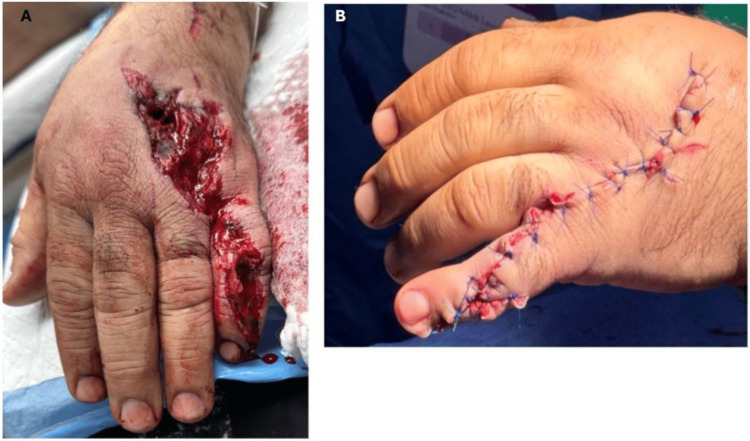
(A) Open laceration involving the dorsal aspect, with extensive soft tissue injury and exposure of the underlying structures from the gunshot wound. The wound margins are irregular, with associated oozing of blood and visible swelling. (B) Left hand post cleansing and debridement and closure

After obtaining informed consent, the patient was brought to the operating room and placed in the supine position. General anesthesia was induced, antibiotics were given, and the patient was intubated. The chest was prepared and draped in a sterile fashion. A midline incision was made along the sternum, extending from the sternal notch to the xiphoid process. The lungs were deflated, the sternum was divided at midline using a powered sternal saw, and the chest cavity was entered. Sternal hemostasis was maintained using bone wax and electrocautery. A self-retaining sternal retractor was placed to provide optimal exposure of the mediastinum and the heart. The pericardium was opened longitudinally, and hemopericardium evacuated. A small penetrating wound was identified on the anterior-superior wall of the right ventricle. There was minimal surrounding tissue disruption, and no active bleeding was noted from the epicardial surface. No additional injuries were identified. The cardiac wound was closed with a Prolene 4-0 figure-of-eight suture. A 24 French (Fr) mediastinal chest tube was then placed anterior to the heart for postoperative drainage and fixed to the skin with Prolene 2-0 suture. Due to the lack of a cardiopulmonary bypass machine in the operating room, the patient was unable to be cannulated for subsequent bypass and bullet removal. The sternum was then approximated using stainless steel wires in a figure-of-eight configuration. The soft tissue layers were then closed in anatomical layers with Vicryl 2-0 in a continuous fashion. The skin was closed with a subdermal Vicryl 3-0 suture and was closed with staples. His left hand was cleansed and debrided by orthopedic surgery and then closed via interrupted sutures. The patient was hemodynamically stable at the conclusion of the procedure and was transferred to the post-anesthesia care unit (PACU) for further monitoring. Intraoperative findings are shown in Figures 3-4.

In the PACU, the patient was hemodynamically stable, off vasopressors, sedated, and under mechanical ventilation. Due to the projectile embolization risk, both cardiothoracic surgery and cardiology were consulted. Cardiothoracic surgery recommended conservative management with serial CXR to monitor bullet location and CT-angiography (CTA) of the chest to better visualize the bullet fragment. Cardiology performed an echocardiogram and found an ejection fraction of 70% and hyperdynamic. The right ventricle was unable to be assessed, and the bullet was unable to be visualized via echocardiogram. Workup with serial troponin was found to be unremarkable.

**Figure 5:**
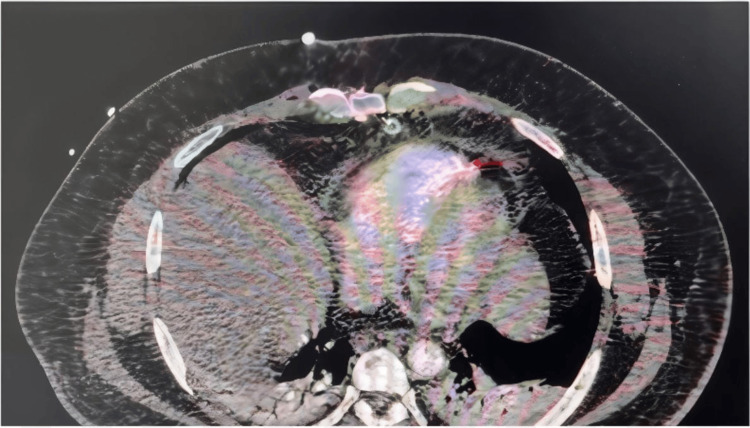
Thoracic computerized tomography-angiogram showing a bullet fragment lodged in the inferior aspect of the right ventricle The bullet fragment pointed by a red arrow in the image. The red arrow pointing to the retained intracardiac bullet fragment in the cardiac apex region.

Follow-up CTA of the thorax revealed that the bullet fragment was localized within the inferior apex of the right ventricle. Imaging is shown in Figure 5.

On postoperative day one, the patient was extubated and transferred to the trauma intensive care unit (TICU). The patient was started on prophylactic anticoagulation with subcutaneous heparin 40 mg every 12 hours for the remainder of his hospital stay. During his stay at the TICU, his vitals remained stable, and follow-up CXRs showed no change in the bullet fragment location, had stable urinary outputs, and had low mediastinal chest tube outputs. His chest pain was minimized with pain management and was otherwise asymptomatic. His left hand underwent further studies and was found to have a left third and fourth metacarpal open fracture. In addition, he had a left small finger distal phalanx open fracture and a left trapezium fracture. Orthopedic surgery performed open treatment with internal fixation of metacarpal fractures with regional anesthesia. A Kirschner wire was used to fix the fractures in retrograde fashion. Another Kirschner wire was used to fix the metacarpal necks to adjacent metacarpals with no complications. The patient returned to the TICU stable, and later the mediastinal tube was removed. On postoperative day three, the patient was transferred to the ward. On postoperative day 10, the patient was discharged home and scheduled for outpatient follow up with trauma surgery, orthopedic surgery, and physical therapy, with no further anticoagulation regimen warranted.

## Discussion

PCI remains one of the most lethal forms of trauma, primarily due to complications such as cardiac tamponade, myocardial infarction, and projectile embolization [13]. In this case, the patient presented with an 8 mm bullet fragment in the right ventricle and an associated hemopericardium but remained hemodynamically stable throughout his hospital stay. Notably, despite a confirmed pericardial effusion on imaging, the patient exhibited no clinical signs of tamponade physiology, including Beck’s triad. Such a presentation is atypical, as tamponade would often be expected in the context of penetrating cardiac trauma with a retained intracardiac projectile.

Although cardiac tamponade is life-threatening, it may paradoxically reduce mortality in PCI by providing temporary hemostasis through pericardial compression [13,14]. The relatively inelastic pericardium can contain bleeding and stabilize patients until definitive surgical intervention is possible. Nonetheless, progressive accumulation of blood can impair diastolic filling and lead to hemodynamic collapse. The absence of tamponade in this patient may reflect a slow rate of pericardial filling or a limited volume of hemorrhage. The interval between the onset of pericardial effusion and the development of tamponade physiology has not been well characterized in the literature.

A second major consideration in this case was the risk of embolization of the retained projectile. Embolization of intracardiac missiles has been documented in both arterial and venous systems, with the potential for severe complications depending on the embolization site [9,10,14-16]. Risk factors include projectile size, velocity, intracardiac location, and hemodynamic status [14-16]. In this case, the bullet was embedded in the apical portion of the right ventricle. While theoretically posing a risk of embolization into the pulmonary arterial system, the patient remained asymptomatic and exhibited no clinical or imaging findings suggestive of embolic events during hospitalization.

Numerous case reports have described missile embolization to distal sites such as the external carotid artery, pulmonary vasculature, and coronary arteries [15-18]. Management strategies for retained intracardiac foreign bodies vary widely, ranging from open surgical extraction and endovascular retrieval to conservative observation [16-18]. In a follow-up series, three patients with retained bullets in the right ventricle were monitored over 25 years; only one required surgical intervention due to tamponade, while the others were successfully managed conservatively and remained asymptomatic [9]. These findings support a case-by-case approach in stable patients, particularly when the projectile is well-seated and not causing immediate complications.

In this case, the patient received prophylactic anticoagulation with subcutaneous heparin 40 mg every 12 hours during hospitalization to reduce the risk of venous thromboembolism, a standard practice in trauma patients with limited mobility or major surgery. While anticoagulation may be considered in patients with retained intracardiac projectiles - particularly in stable patients managed conservatively [19,20] - the decision must be individualized based on clinical status, projectile location, and bleeding risk. In this patient, the bullet fragment was securely embedded in the trabeculated apex of the right ventricle, with no evidence of systemic embolism, arrhythmia, or instability. Moreover, the absence of cardiopulmonary bypass, recent cardiac repair, and a lack of outcome data supporting extended anticoagulation argued against discharge therapy. Instead, in-hospital prophylaxis was deemed sufficient, with planned outpatient follow-up to monitor delayed thromboembolic events. This approach reflects current best practices in balancing thrombotic and hemorrhagic risks in the absence of guideline-directed management [19,20].

## Conclusions

This case underscores the clinical complexity inherent in the management of penetrating cardiac injuries, particularly those involving retained intracardiac projectiles. It highlights the paradoxical role of cardiac tamponade in stabilizing patients by limiting hemorrhage and the importance of nuanced interpretation of imaging and physical exam findings. Although hemopericardium and a retained right ventricular bullet were present, the patient remained stable and underwent successful repair via median sternotomy and pericardiotomy. The bullet fragment was not removed due to the lack of cardiopulmonary bypass availability and the absence of symptoms or migration. Prophylactic anticoagulation was administered, and the patient completed a stable hospital course without complications. This case supports a conservative, individualized approach in select patients and underscores the need for further research to establish evidence-based guidelines for managing retained intracardiac foreign bodies.
